# Do place of residence and ethnicity affect health services utilization? evidence from greece

**DOI:** 10.1186/1475-9276-10-16

**Published:** 2011-04-26

**Authors:** Eleni Lahana, Evelina Pappa, Dimitris Niakas

**Affiliations:** 1 Faculty of Social Sciences, Hellenic Open University, Patras, Greece; 2 Faculty of Nursing, Technological Educational Institute of Larissa, Greece

## Abstract

**Background:**

Equal utilization of health services for equal need, is one of the main targets for public health systems. Given the public-private structure of the Greek NHS, the main aim of the study was to investigate the impact of underlying factors, such as health care needs, socio-demographic characteristics and ethnicity, on the utilization of primary and hospital health care in an urban and rural population of the Greek region, Thessaly.

**Methods:**

A cross-sectional study was carried out in 2006 in Thessaly, a Greek region of Central Greece, in a representative sample of 1372 individuals (18+ years old, response rate 91.4%) via face-to-face interview. Health care needs were determined by self-perceived health status estimated by the SF-36 Health Survey, using the summary scores of physical and mental health. The utilization of primary care was measured by last month visits to 1) primary public services and 2) private practitioners visits and utilization of secondary care was measured by past year visits to 3) public hospital emergency departments and 4) admissions to public hospitals. Multivariable stepwise logistic regression analysis was applied in the whole sample and separately for the urban and rural population, in order to determine the predictors of health services utilization. Statistical significance was determined with a p value < 0.05.

**Results:**

Health care needs were the most significant determinants of primary and secondary health services utilization in both the urban and rural areas. Poor physical and mental health was associated with higher likelihood of use. In the urban areas middle-aged, elderly and Greeks were more likely to use primary health services, whereas primary education was associated with more visits to the emergency departments. Wealthier individuals were two times more likely to be admitted to hospitals. Individuals from the rural areas with university education visited more the public primary services, while wealthier individuals visited more the private practitioners. Immigrants had a higher likelihood of visiting emergency departments.

**Conclusions:**

Although health care needs were the main determinant of health services utilization in both the urban and rural population, socio-economic and ethnic differences also seem to contribute to the inequities observed in some types of health services use, favouring the better-off. Such findings provide important information to policy makers, which attempt to reduce inequalities in health care according to place of residence and ethnicity.

## Background

Equal health services utilization for equal need is one of the main targets for public health systems and it is also an attractive issue for research, since health care inequalities are present in every health system [1] and have been extensively studied during the last three decades. The main focus of attention has been the horizontal inequity between differential demographic, socio-economic and geographic subgroups [1,2]. The usual approach has been the determination of health care reception in accordance to health need and the investigation of how "unjust" factors determine utilization.

According to the behavioral model [3,4] there is equitable access when predisposing and need factors determine the use, whereas inequity is present when enabling factors determine the use. Many studies have shown that all of the above factors have been associated with utilization of health care services and their impact varies with the type of health service. Female gender and older age are related to increased use [5-10]. Socio-economic status is also found to be a significant predictor of health care utilization and is a strong determinant of health inequalities [11-14]. Furthermore, place of residence and ethnicity are two factors that not only determine the populations' characteristics and its social structure, but they also define the differential access to health care services [15-21]. Finally, several studies have identified that the estimation of perceived health status or health-related quality of life (HRQOL) are important determinants of health care use and they can predict health service utilization [6,22-25].

Previous studies in Greece [22,26] have shown that utilization of health services is mostly influenced by health need factors, such as self-perceived health, whereas socio-economic factors have a mediocre effect, indicating therefore a pro-poor inequality when visiting a physician. Place of residence and ethnicity are two factors that have not been previously studied in Greece. Accessibility of health care services according to place of residence is of primary importance, especially in rural areas, since rural dwellers encounter many barriers, such as long travelling distances when accessing health care services [27,28]. On the other hand, ethnic diversity in the Greek society differentiates the ability of all groups to access health care and is of great importance for public health. It is therefore important to know that the majority of immigrants (88%) in the area of Thessaly are of Albanian ethnicity.

However the most striking issue is that the majority of previous studies examine the regional differences in health care use between urban and rural residents and little attention has been given when investigating differences in health care use within urban and rural areas. Rural and urban areas differ in respect to demographic composition, distribution of socioeconomic indices and provision of health care resources. Socioeconomic inequalities and horizontal inequity in health care use within urban and rural areas are issues of great importance and should be extensively studied.

The aim of this study was to investigate the use of primary and secondary health services in a Greek urban and rural population. We attempted to determine whether demographic, socioeconomic and health needs determine health care use and to quantify their impact if any. Also, we tried to identify the different utilization patterns in respect to place of residence and ethnicity.

### The Greek Health Care System

The Greek health care system is a mixed public-private health care system, which provides full public coverage to the entire population, whereas at the same time an over-developed private sector is also present and plays an important role in the structure of the health system. Health care services in Greece are basically provided by i) the National Health System (public hospitals and health centres in rural and semi-urban areas), ii) the health insurance funds (health centres with salaried physicians or contracted private physicians) and iii) the private sector (hospitals, diagnostics centres, private practitioners) that require out of pocket payments at the point of use. Health insurance funds are public schemes financed by employees, employers and the public budget. In Greece it is mandatory for the entire workforce (including their families) to be insured according to professional status by one of the 8 different health insurance funds.

Health care use of primary health services in Greece is highly dependable upon the place of residence, since the rural population is mostly covered by the Health Centres (17 in total in the area of Thessaly), which provide public primary care by salaried specialized doctors and the rural medical practices (160 in total, staffed with unspecialized doctors). On the other hand, in the urban regions public primary health care is also provided by the public health centres, but in addition care is also provided by the insurance funded health centres (4 central and 7 suburban) and the insurance covered private practices [29]. In addition, private doctors that require out of pocket payments are also used by individuals that are better-off in economic terms, which are mainly situated in urban regions. Primary care is mainly provided by the specialised doctors, provided by all of the above types of services that are approached by the patient without referral by a general practitioner. There are 3.388 private practices covering a population of 753.888 in the region of Thessaly. Additionally, secondary health care is provided by the public hospitals and hospital emergency departments found in the four central urban centres of Thessaly, like Larissa, Trikala, Volos and Karditsa and their use is free of charge. Public hospitals offer approximately 2.5 beds per 1000 residents and are mainly used by the population of the region. Urban residents and particularly immigrants seem to visit emergency department's more than their rural counterparts [30]. Secondary care though is also provided by private hospitals that require payment but these do not offer emergency departments.

## Materials and methods

### Study and data

The study included a stratified sample of residents in the geographic region of Thessaly. Thessaly is one of the 13 geographic regions of Greece, subdivided into four prefectures and is also the third largest region, population-wise, where 6.7% of the Greek population lives. The population of Thessaly is considered to be one of the most representative samples for health care utilization in the mainland of Greece. The participants in the study were chosen proportionally to the population size, according to a three-staged methodology. In particular, in the first stage a random sample of building blocks was selected according to the information collected from the 2001 national census and the immigration office. In the second stage, households were selected from every block by systematic sampling and in the third stage one participant (>18 years old) was selected from each household by simple random sampling. In total, 1372 residents i.e. 1042 Greeks and 330 Albanians out of the 1500 initially approached (response rate 91.4%) agreed to participate, constituting a representative sample of the population living in this particular region. Institutionalized individuals were excluded.

The questionnaires answered by the individuals via face to face interview, included questions concerning the demographic and socio-economic characteristics, and also questions found in the previously validated SF-36 Health Survey [31,32] in an attempt to estimate self perceived health related quality of life, in addition to questions that could provide information on health service utilization. Oral informed consent was obtained from all the participants before the interview and the study was also approved by the ethics committee of the Hellenic Open University.

### Measurements

The main dependent variables involved in the study were assessed by the utilization of the following four types of services: i) public health centres or insurance funded private or public physicians and ii) private practitioners, during the last month and iii) emergency departments of public hospitals together with iv) hospital admissions, during the last year.

Based on the behavioural model several independent variables were selected in order to create a model that would sufficiently explain the health care utilization patterns of the participants. In particular, predisposing characteristics like gender, age (six age categories) and ethnicity (Greeks/immigrants) were selected as determinants of health care utilization. Furthermore, enabling factors that facilitate or impede use including educational level (divided to three levels primary, secondary and university), monthly household income (divided to low < 880 €, medium 880.01-1760 € and high >1760.01 €) and occupation (manual/non manuals) were also estimated, together with the level of urbanization (rural/urban place of residence).

Need for care was proximately addressed by self-perceived health status, as measured by the two SF-36 summary scores of physical (PCS) and mental (MCS) health. Higher component scores for PCS and MCS reflect better perceived health. The use of summary scores provides the advantage of requiring fewer statistical comparisons when analysing SF-36 data without substantially losing the information provided by the questionnaire. Recent studies in Greece [31,32] validated the Greek SF-36 Health Survey questionnaire and their results were comparable with previous studies.

### Statistical analysis

The selected independent and dependent variables described above were included in the model of health care utilization and the differences in physical and mental health across the socio-demographic characteristics were assessed by the student t-test and ANOVA in a univariate analysis. All the variables were tested for linear association with Pearson's correlation and no significant associations were found. In addition, multivariable stepwise logistic regression analysis was conducted to determine the predictors of health services utilization. Four logistic regression models were applied and the exponentiation of B coefficient Exp(b) was used in order to estimate the adjusted odds ratio (OR) for each independent factor, with 95% confidence intervals. This analysis was also applied separately for the urban and rural populations. Results were considered statistically significant with a p value < 0.05 and all the statistical analysis was performed with the statistical package SPSS v15.0.

## Results

### Prevalence of health care use

Our sample consisted 48% males and 52% females that approximately represented the gender distribution of the population in Thessaly according to the 2001 national census. The overall mean age was 43 years and the individuals were sub-divided into five age categories. Table 1 provides information on the sample distribution according to socio-demographic characteristics and the selected health care utilization variables. Utilization in all types of services, primary and secondary, was approximately the same regardless of the place of residence, rural or urban. The rural population though reported higher use of insurance funded physicians (22.5%), whereas the urban population reported higher use of the hospital emergency departments (29%) and more admissions to public hospitals (14%).

**Table 1:** Distribution of the urban and rural population according to their socio-demographic characteristics and the selected health care use variables

	GENERAL POPULATION	URBAN	RURAL
	(n = 1372)	(n = 906)	(n = 466)
**Gender**	**n (%)**	**n (%)**	**N (%)**
Male	660 (48.1)	441 (48.6)	219 (47.0)
Female	712 (51.9)	465 (51.3)	247 (53.0)

**Age**			
18-34	431 (31.4)	290 (32.0)	141 (30.3)
35-44	378 (27.6)	240 (26.5)	136 (29.2)
45-54	256 (18.7)	163 (18.0)	93 (20.0)
55-64	152 (11.1)	106 (11.7)	46 (9.8)
65-75+	155 (11.3)	106 (11.7)	49 (10.5)

**Residence**			
Rural	466 (34.0)		
Urban	906 (66.0)		

**Income**			
Low	398 (29.0)	252 (27.8)	146 (31.3)
Medium	692 (50.4)	460 (50.7)	232 (49.7)
High	282 (20.6)	194 (21.4)	88 (18.8)

**Education**			
Primary	355 (25.9)	230 (25.3)	125 (26.8)
Superior	819 (59.7)	550 (60.7)	269 (57.7)
University	198 (14.4)	126 (13.9)	72 (15.4)

**Occupation**			
manual	805 (58.7)	469 (51.7)	336 (72.1)
Non manual	567 (41.3)	437 (48.2)	130 (27.9)

**Ethnicity**			
Greeks	1042 (75.9)	649 (71.6)	393 (84.3)
Albanians	330 (24.1)	257 (28.3)	73 (15.6)

Physician of Insurance Funds*			
Yes	297 (21.6)	192 (21.2)	105 (22.5)
No	1075 (78.4)	714 (78.8)	361 (77.5)

**Private Practitioner**			
Yes	222 (16.2)	145 (16.0)	77 (16.5)
No	1150 (83.8)	761 (84.9)	389 (83.5)

**Emergency Departments**			
Yes	375 (27.3)	264 (29.1)	110 (23.6)
No	998 (72.7)	778 (85.9)	356 (76.4)

**Hospitals**			
Yes	187 (13.3)	128 (14.1)	58 (12.4)
No	1186 (86.4)	642 (70.9)	408 (87.6)

PCS = Physical Component Score. MCS = Mental Component Score

* In rural and semi-urban areas public primary care is provided by health centres.

In the univariate analysis (table 2), the mean differences in the physical and mental health summary scores between and within the subgroups that are determined by the socio-economic status and demographic characteristics, revealed that education, income and age were the most significant predictors. In detail, the older, less educated and poorer individuals reported lower scores for physical and mental health, although the deterioration in physical health appeared to be more rapid, since mental health was not significantly different in the older and poorer rural residents. Gender and occupation (with some exceptions) together with ethnicity did not show any statistical significance, something that was also observed in the urban and rural subgroups.

**Table 2:** Mean physical and mental health summary scores of individuals utilizing health care (Users) stratified by socioeconomic characteristics in the whole sample and in the sample from the urban and rural regions.

		PCS (CI)	MCS(CI)				
**Non users**		52.4 (48.1 - 58.4)	46.8 (42.4 - 53.3)				
**Users**		47.3 (43.0 - 53.2)	41.3 (37.0 - 47.8)				
Sig.*		p < .001	p < .05				

		**USERS WHOLE SAMPLE**		**USERS URBAN**		**USERS RURAL**	
		**PCS (CI)**	**MCS(CI)**	**PCS (CI)**	**MCS(CI)**	**PCS (CI)**	**MCS(CI)**

**Sex**							
	**Male**	47.5 (46.5-48.5)	42.7 (41.6-43.8)	47.6 (46.8-50.2)	42.6 (43.3-47.2)	47.4 (44.6-49.5)	42.8 (42.6-48.4)
	**Female**	47.0 (46.1-48.0)	40.1 (39.0-41.2)	46.7 (45.9-49.3)	40.0 (40.7-44.6)	47.6 (44.9-49.8)	40.2 (39.9-45.8)
	Sig.*	N.S.	p < .05	N.S.	p < .05	N.S.	N.S.

**Age**							
	**18-34**	53.1 (52.00-54.01)	43.3 (41.05-45.02)	53.0 (51.8-54.3)	44.4 (42.1-46.8)	53.1 (51.2-55.1)	41.3 (37.7-44.9)
	**35-44**	49.9 (48.9-51.0)	41.9 (40.6-43.1)	50.1 (48.8-51.4)	41.5 (39.9-43.0)	49.5 (47.6-51.4)	42.6 (40.1-45.1)
	**45-54**	47.1 (45.8-48.4)	41.4 (39.9-43.0)	47.2 (45.5-48.9)	41.9 (39.9-44.0)	47.0 (44.7-49.2)	40.5 (38.0-42.9)
	**55-64**	42.3 (40.6-43.9)	39.3 (37.2-41.3)	42.0 (40.0-44.0)	38.5 (36.1-40.9)	43.0 (39.0-47.0)	41.4 (36.1-46.7)
	**65-75+**	38.3 (36.9-39.8)	39.1 (36.9-41.3)	38.1 (36.1-40.1)	38.4 (35.4-41.4)	38.8 (36.0-41.5)	40.4 (36.2-44.7)
	Sig.*	p < .001	p < .05	p < .001	p < .05	p < .001	N.S.

**Residence**							
	**Rural**	47.5 (46.3-48.7)	41.4 (40.0 -42.8)				
	**Urban**	47.2 (46.3-48.0)	41.3 (40.4-42.3)				
	Sig.*	N.S.	N.S.				

**Income**							
	**Low**	43.9 (42.6-45.2)	39.1 (37.6-40.5)	43.1 (41.4-44.9)	38.3 (36.4-40.1)	45.2 (42.8-47.6)	40.5 (37.8-43.3)
	**Medium**	47.9 (47.0-48.7)	41.74 (40.6-42.8)	48.3 (47.2-49.3)	42.2 (40.9-43.6)	47.0 (45.3-48.7)	40.5 (38.6-42.5)
	**High**	50.96 (49.5-52.3)	43.9 (42.1-45.7)	50.1 (48.2-52.0)	43.4 (41.2-45.5)	52.6 (50.5-54.7)	44.9 (41.3-48.5)
	Sig.*	p < .001	p < .001	p < .001	p < .001	p < .001	N.S.

**Education**							
	**Primary**	41.1 (39.9-42.3)	39.2 (37.8-40.6)	40.8 (39.2-42.3)	39.0 (37.0-41.0)	41.7 (39.5-43.9)	39.6 (37.0-42.1)
	**Superior**	49.5 (48.7-50.3)	41.7 (40.7-42.6)	49.4 (48.4-50.3)	41.9 (40.8-43.1)	49.7 (48.3-51.2)	41.0 (39.1-42.9)
	**University**	51.4 (49.9-52.9)	44.9 (42.4-47.6)	51.1 (49.1-53.1)	43.9 (40.4-47.3)	51.9 (49.4-54.4)	46.4 (42.1-50.8)
	Sig.*	p < .001	p < .001	p < .001	p < .05	p < .001	p < .05

**Occupation**							
	**manual**	46.7 (45.7-47.6)	40.8 (39.6-41.9)	45.9 (42.0-45.4)	40.5 (37.1-41.0)	47.7 (45.7-50.9)	41.1 (37.1-43.4)
	**non manual**	48.00 (47.0-48.9)	42.0 (41.0-43.1)	48.2 (44.2-47.7)	42.0 (38.6-42.5)	47.1 (45.1-50.3)	42.0 (38.0-44.3)
	Sig.*	N.S.	N.S.	p < .05	N.S.	N.S.	N.S.

**Ethnicity**							
	**Greeks**	47.0 (46.2-47.9)	41.3 (40.3-42.3)	46.7 (43.4-47.0)	41.3 (39.1-43.3)	47.6 (45.0-51.4)	41.3 (37.2-44.9)
	**Albanians**	47.9 (46.8-49.0)	41.4 (40.2-42.6)	48.1 (44.9-48.5)	41.4 (39.2-43.4)	47.0 (44.4-50.8)	41.6 (37.5-45.2)
	Sig.*	N.S.	N.S.	N.S.	N.S.	N.S.	N.S.

### Determinants of health care utilization

Table 3 displays the results that were revealed by the regression models as far as the whole sample is concerned. The variable that most strongly associated with all the types of health services under study was self-perceived health. Individuals reporting poorer physical and mental health status were more likely to use insurance funded physician services, doctors from the private sector or hospitals by visits to emergency departments and admissions to public hospitals. Furthermore, a clear gradient was found within the different levels of education and income as far as primary health care services use was concerned. Individuals with university education had a higher likelihood (Exp(b) 2.33) of visiting an insurance funded physician than those with primary education, whereas individuals with higher income were more likely (Exp(b) 1.83) to visit a private doctor. Ethnicity was a significant predictor of the utilization of insurance funded doctors and hospital emergency departments, with Greeks being more likely to visit an insurance funded physician (Exp(b) 1.97) and less likely to be admitted to emergency departments (Exp(b) 0.39). Men and non-manual workers were less likely to be admitted to emergency departments. Finally, middle age was associated with greater probability of visiting doctors that are in the public or the private sector, whereas the elderly were more likely to visit doctors covered by the insurance funds.

**Table 3:** Logistic regression models for the General Population

	Visits to insurance funds physicians			Visits to private practitioners			Admissions to emergency departments			Hospital admissions		
	**P-value**	**Exp(b)**	**CI**	**P-value**	**Exp(b)**	**CI**	**P-value**	**Exp(b)**	**CI**	**P-value**	**Exp(b)**	**CI**

**Sex (men)**	NS	1.08	0.79-1.48	NS	0.86	0.62-1.20	<0.05	1.39	1.06-1.88	NS	1.28	0.89-1.86

**Age (18-34)**												
**35-44**	NS	1.32	0.85-2.05	NS	1.33	0.85-2.06	NS	1.12	0.79-1.61	NS	1.02	0.61-1.71
**45-54**	<0.04	1.81	1.12-2.91	<0.05	1.71	1.05-2.78	NS	0.82	0.54-1.26	NS	0.72	0.39-1.31
**55-64**	NS	1.22	0.67-2.23	NS	0.99	0.51-1.92	NS	0.91	0.53-1.56	NS	1.07	0.54-2.10
**65+**	<0.001	3.11	1.65-5.88	NS	1.31	0.62-2.76	NS	0..99	0.54-1.82	NS	1.19	0.55-2.53

**Ethnicity (Greeks)**	<0.01	1.97	1.27-3.05	NS	0.89	0.60-1.32	<0.001	0.39	0.28-0.53	NS	0.83	0.53-1.30

**Education (primary)**							NS					
**Secondary**	NS	1.10	0.72-1.69	NS	1.21	0.76-1.93	NS	0.73	0.50-1.09	NS	0.96	0.58-1.58
**University**	<0.01	2.33	1.33-4.06	NS	1.11	0.59-2.09	NS	0.60	0.34-1.03	NS	1.16	0.57-2.33

**Income (low)**												
**Medium**	NS	0.71	0.50-1.01	NS	1.45	0.97-2.17	NS	1.15	0.82-1.60	NS	1.08	0.70-1.66
**High**	NS	1.89	0.56-1.43	<0.05	1.83	1.10-3.04	NS	1.27	0.83-1.95	NS	1.52	0.86-2.67

**Occupation (non manual)**	NS	0.81	0.59-1.11	NS	1.90	0.64-1.26	<0.05	0.74	0.56-0.98	NS	0.69	0.47-1.00

**PCS**	<0.001	0.93	0.91-0.95	<0.01	1.96	0.94-0.98	<0.001	0.95	0.93-0.96	<0.001	0.91	0.89-0.93

**MCS**	<0.001	0.95	0.94-0.97	<0.001	1.94	0.93-0.96	<0.001	0.96	0.95-0.97	<0.001	0.96	0.94-0.98

**R**^**2**^	22.5			11.1			15.6			17.2		

PCS = Physical Component Score, MCS = Mental Component Score

NS = non significant (p > 0.05)

For categorical explanatory variables, the reference group for the calculation of the odds ratio (OR) is indicated by the parenthesis.

In the area-specific analysis (table 4 and 5), urban and rural residents exhibited different utilization patterns of the four types of health care. Health status remained the most significant determinant of health services utilization in both the urban and rural areas, with higher scores reflecting lower likelihood of use.

**Table 4:** Logistic regression models for the Urban Population

	Visits to physicians of insurance funds			Visits to private practitioners			Admissions to emergency departments			Hospital admissions		
	P-value	Exp(b)	CI	P-value	Exp(b)	CI	P-value	Exp(b)	CI	P-value	Exp(b)	CI
**Sex (men)**	NS	1.04	0.70-1.55	NS	0.94	0.62-1.41	<0.05	1.47	1.06-2.05	NS	1.05	0.67-1.65

**Age (18-34)**							NS					
**35-44**	NS	1.42	0.81-2.48	<0.05	1.73	1.00-3.01	NS	1.10	0.71-1.69	NS	1.32	0.70-2.48
**45-54**	<0.05	2.00	1.09-3.66	<0.05	2.01	1.07-3.75	NS	0.83	0.49-1.39	NS	0.73	0.34-1.58
**55-64**	NS	1.11	0.52-2.35	NS	1.50	0.67-3.34	NS	0.95	0.50-1.78	NS	1.26	0.55-2.89
**65+**	<0.01	2.97	1.33-6.60	NS	1.37	0.52-3.59	NS	0.80	0.38-1.67	NS	1.80	0.71-4.60

**Ethnicity (Greeks)**	<0.05	1.96	1.18-3.28	NS	0.77	0.48-1.24	<0.001	0.36	0.25-0.54	NS	0.63	0.37-1.07

**Education (primary)**												
**Secondary**	NS	1.06	0.62-1.80	NS	1.50	0.83-2.72	<0.05	0.57	0.35-0.92	NS	0.93	0.50-1.72
**University**	NS	1.53	0.74-3.14	NS	1.24	0.54-2.83	<0.05	0.47	0.24-0.92	NS	0.79	0.32-1.96

**Income (low)**							NS					
**Medium**	NS	0.79	0.50-1.23	NS	1.23	0.74-2.03	NS	1.16	0.78-1.74	NS	1.18	0.69-2.03
**High**	NS	0.81	0.45-1.47	NS	1.38	0.73-2.59	NS	1.30	0.78-2.16	<0.05	2.06	1.04-4.08

**Occupation (non manual)**	NS	0.82	0.55-1.23	NS	0.79	0.51-1.21	NS	0.81	0.57-1.15	NS	0.85	0.53-1.35

**PCS**	<0.001	0.94	0.91-.096	NS	0.97	0.94-1.00	<0.001	0.95	0.92-0.97	<0.001	0.91	0.88-0.94

**MCS**	<0.001	0.95	0.94-0.97	<0.001	0.94	0.92-0.96	<0.01	0.97	0.95-0.98	<0.01	0.97	0.94-0.99

**R**^**2**^	21.3			11.2			14.7			18.5		

PCS = Physical Component Score, MCS = Mental Component Score

NS = non significant (p > 0.05)

For categorical explanatory variables, the reference group for the calculation of the odds ratio (OR) is indicated by the parenthesis.

**Table 5:** Logistic regression models for the Rural Population

	Visits to doctors of insurance funds			Visits to private doctors			Admissions to emergency departments			Hospital admissions		
	P-value	Exp(b)	CI	P-value	Exp(b)	CI	P-value	Exp(b)	CI	P-value	Exp(b)	CI
**Sex (men)**	NS	1.19	0.69-2.05	NS	0,69	0,38-1,23	NS	1,33	0.80-2.19	NS	1.81	0.92-3.55

**Age (18-34)**										NS		
**35-44**	NS	1.25	0.59-2.67	NS	0.85	0.39-1.84	NS	1.21	0.63-2.32	NS	0.68	0.26-1.74
**45-54**	NS	1.68	0.76-3.72	NS	1.39	0.63-3.09	NS	0.78	0.36-1.69	NS	0.71	0.26-1.91
**55-64**	NS	1.58	0.56-4.45	NS	0.33	0.08-1.28	NS	0.83	0.29-2.38	NS	0.76	0.22-2.65
**65+**	<0.01	4.24	1.41-12.6	NS	1.05	0.30-3.63	NS	1.64	0.55-4.83	NS	0.51	0.13-2.02

**Ethnicity (Greeks)**	NS	1.94	0.76-4.94	<0.05	2.55	1.01-6.41	<0.01	0.35	0.18-0.66	NS	1.41	0.47-4.16

**Education (primary)**												
**Secondary**	NS	1.38	0.64-3.00	NS	0.79	0.36-1.76	NS	1.32	0.64-2.71	NS	0.91	0.36-2.30
**University**	<0.01	5.33	2.04-13.9	NS	0.71	0.25-2.02	NS	1.08	0.39-3.00	NS	1.90	0.57-6.28

**Income (low)**												
**Medium**	NS	0.50	0.29-1.02	<0.05	2.26	1.10-4.64	NS	0.98	0.54-1.81	NS	0.91	0.43-1.95
**High**	NS	1.17	0.53-2.59	<0.01	4.17	1.69-10.2	NS	1.15	0.51-2.56	NS	0.89	0.29-2.71

**Occupation (non manual)**	NS	0.78	0.44-1.37		1.63	0.85-3.14	<0.05	0.58	0.35-0.98	<0.05	0.49	0.25-0.96

**PCS**	<0.001	0.91	0.88-0.95	<0.001	0.93	0.89-0.96	<0.01	0.94	0.91-0.97	<0.001	0.90	0.86-0.94

**MCS**	<0.001	0.94	0.92-0.97	<0.001	0.94	0.92-0.97	<0.001	0.94	0.92-0.97	<0.01	0.95	0.93-0.99

**R**^**2**^	27.9			19.9			18.4			19.1		

PCS = Physical Component Score, MCS = Mental Component Score

NS = non significant (p > 0.05)

For categorical explanatory variables, the reference group for the calculation of the odds ratio (OR) is indicated by the parenthesis.

### Urban areas

In the urban areas (table 4), age was related to the utilization of primary services, with the middle-aged being 2 times more likely to visit an insurance funded physician (exp(b) 2.00) or a private doctor (exp(b)2,01) and the elderly (65+) being three times more likely to visit an insurance funded physician (exp(b) 2.97). Ethnicity was also associated with visits to insurance funded primary care health services and admissions to the public hospitals' emergency departments. Greeks were 97% more likely to use insurance funded services than immigrants and 64% less likely to be admitted to emergency departments. Additionally, individuals with secondary or university education had a lower likelihood of using emergency departments compared to those with primary education. Finally, it was found that wealthier individuals were two times more likely to be admitted to hospitals (exp(b) 2.06).

### Rural areas

In the rural areas (table 5), ethnic differences determined the use of doctors in the private sector and the visits to emergency departments, with Greek individuals being more than two times likely to visit a private practitioner, that requires and out of pocket payment and about 65% less likely to be admitted to emergency departments. Furthermore, individuals with university education were more than five times likely to visit insurance funded doctors in health centers (Exp(b) 5.33), whereas wealthier individuals (with medium or high income) had a higher likelihood of visiting a private doctor. Finally, non-manual workers were less likely than manual workers to be admitted to emergency departments (Exp(b) 0.58) and to hospitals (Exp(b) 0.49).

## Discussion

The present study revealed that health services utilization is mostly related to health needs estimated by self-perceived physical and mental health, an effect that was also evident in the sub-analysis of both the urban and rural population. It has been previously shown that there is equitable use of health services when demographic characteristics or need factors determine the use [3,4]. However, in many instances the role of the socio-economic status was found to be a very important determinant of health care utilization, which implies the existence of clear socio-economic gradients.

Our findings indicated a consistent association between health care utilization and health needs, shown by the association of impaired physical and mental health with the use of primary or secondary health services. These findings are in accordance to the results of previous studies [33-36]. Self-perceived health is a good predictor of health needs and the SF-36 summary scores for physical and mental health have been previously used in many studies as reliable estimating factors of self perceived health [22,37]. Our study also confirmed that these summary scores predicted well the use of all types of health services that were included in this study.

Furthermore, it was very interesting to observe that gender differences were not that evident, with the exception that female urban residents visited the emergency department more, as opposed to a previous study in Greece [22], where clear gender differences in utilization of primary health services were evident. Gender differences in health care use are well-known and studies addressing health care and morbidity have demonstrated that women have worse perception of health status than men and are more likely to use health services [5,6,8,36], while men seem to visit health services when health problems become urgent, therefore reporting higher hospitalization rates [8].

Another important finding was the existence of socio-economic inequalities in health care use within urban and rural areas. General health care utilization studies have shown that there is a pro-poor inequality in General Practitioner (GP) utilization and hospital admissions [22,23]. This utilization pattern differed totally when studying utilization disparities within a particular geographic region. In rural areas, our results showed a significant social (educational) gradient, a finding that has been corroborated by a previous study [38], where highly educated individuals visit more the doctors provided by public health centers. This pattern of utilization is probably due to the fact that health centers constitute the basic public provider of primary care in rural populations, after controlling for health needs.

The above obvious horizontal inequity is possibly explained by the following aspects. On the supply side, the state of rural health services in Greece is poor [39] and the scarcity of resources increase the unequal use. According to a recent study [39] there are significant shortages of medical and nursing staff and the essential equipment is limited in the majority of health centers in the rural setting. Furthermore, rural residents have to overcome certain obstacles when trying to access health services, like longer travelling distances and lack of transportation [17,40], especially in remote places. These constrains are expected to have an effect upon the most vulnerable individuals i.e individuals of a low socio-economic status. On the demand side, individuals with higher education seem to be more motivated to seek opportunities and have the skills to benefit from primary health care more effectively than those of a low educational level.

As expected, wealthier individuals visited more doctors from the private sector, since these individuals can afford the out of pocket payments required for the private sector consultations. This difference in wealthy individuals is also indicative of the fact that a significant portion of rural residents is able to seek private primary health care services and they don't rely on the long waiting lists of public services. The association between income and use of private physicians is well-known [1], and this finding implies that real income-related inequities could be masked by the fact that wealthier rural residents cover their possible unmet health needs from the private sector. It is therefore observed that utilization of primary health care services is mostly affected in rural areas, since secondary care in these areas is scarce and can be accessed in bigger urban centers.

Contrarily, in urban areas our results showed that income is a significant determinant of secondary care use after adjustment of health need, indicating a clear gradient. Previous studies have shown a pro-poor inequality in hospitalization, whereas a previous Greek study [22] showed that hospitalization was associated only with health need and that the socio-economic gradient did not have a statistically significant effect. The present results may be indicative of the fact that wealthier individuals have inappropriate high rates of hospital admissions or that poor individuals have low utilization because of the pro-rich inequity that is formed in health services use. However, further studies are needed in order to elucidate the causative factors of inequity in hospital admissions especially when taking into account that provision of secondary care in Greece through the NHS secures free access but possibly with lower quality.

The above data can offer important information on the inequities formed by enabling factors that are significant for policy makers confronting inequalities between rural and urban geographical areas, in order to ensure equity in health.

Ethnic groups on the other hand, exhibited differential utilization patterns according to the type of health services. In particular, Greeks were more likely to visit public primary health care, whereas Albanians were more likely to visit hospital emergency departments, a finding also observed in a previous study [20,21,41]. It becomes obvious that immigrants replace primary health care with emergency departments, possibly due to their restricted access to insurance funded primary care. Findings from previous studies suggest that health care insurance is a critical cause of differences between immigrants and non immigrants as far as access to primary care is concerned [42]. Another possible explanation of the reduced utilization of primary health care in immigrants could be the fact that immigrants in our study were of a younger age therefore they were less likely to report health problems unless it was an emergency. Immigrants in Greece are mostly occupied in manual occupations, so they are at a higher risk of accidents and more likely to be submitted to hospital emergency departments. Therefore, immigrants seek treatment at the emergency departments of public hospitals that is free at the point of use and is highly accessible to this ethnic group that is mostly situated in an urban setting.

There were some limitations in our study. Utilization of health services is known to include two components∙ the initial use and the frequency of use. In our study the frequency of use was not addressed, due to unavailability of data. Furthermore, the selection of the independent variables structuring our utilization model was based on the behavioral model and it is important to include in studies of health care use other variables, such as health behaviors, chronic diseases and individuals' expectations. Lastly, this survey measured health services utilization based on self-reported data and as it is well known such data are subjected to measurement errors that arise when respondents are asked to recall past utilization [43], so the presented results should be interpreted under the light of this limitation.

## Conclusions

Our study shows that socio-economic inequities in health care use are present between and within different geographic areas, whether urban or rural, but these inequities appeared to be stronger in the rural areas. Horizontal inequity indicated that health care use favours the better-off in both the urban and rural areas. Being of a higher educational level or income could increase pro-rich inequity especially when primary health care of rural areas is concerned, even after controlling for health needs, whereas income-related inequities, favouring wealthier residents, were also present in the urban areas, as far as hospital admissions were concerned. Ethnic groups experienced higher degrees of inequity in primary health care that is possibly caused by their restricted access in social insurance health care.

The distribution of health and health services within regions and between subgroups are equally important. Such findings provide information for policy makers to improve equity in health care. Variations in the inequities of health care as determined by place of residence points to more explicit targets for policy measures and resource allocations within the settings of urbanity and rurality.

## Competing interests

The authors declare that they have not competing interests.

## Authors' contributions

EL has participated in the design of the study, involved in the statistical analysis and drafted the manuscript, EP helped in the statistical analysis and interpreted the results and DN conceived the study, interpreted the results and made refinements of the manuscript for intellectual content. All authors have read and approved the final manuscript.
